# The early days of plastid retrograde signaling with respect to replication and transcription

**DOI:** 10.3389/fpls.2012.00301

**Published:** 2013-01-03

**Authors:** Kan Tanaka, Mitsumasa Hanaoka

**Affiliations:** ^1^Chemical Resources Laboratory, Tokyo Institute of TechnologyYokohama, Japan; ^2^Graduate School of Horticulture, Chiba UniversityMatsudo, Japan

**Keywords:** cell cycle, checkpoint, chloroplast, DNA replication, nuclear gene transfer, retrograde signal, tetrapyrrole, transcription

## Abstract

The plastid signal was originally defined as a pathway that informs the nucleus of the chloroplast status and results in the modulation of expression of nuclear-encoded plastid protein genes. However, the transfer of chloroplast genes into the nuclear genome is a prerequisite in this scheme, although it should not have been established during the very early phase of chloroplast evolution. We recently demonstrated in a primitive red alga that the plastid-derived Mg-protoporphyrin IX activates nuclear DNA replication (NDR) through the stabilization of a G1 cyclin, which coordinates the timing of organelle and NDR. This mechanism apparently does not involve any transcriptional regulation in the nucleus, and could have been established prior to gene transfer events. However, a retrograde signal mediating light-responsive gene expression may have been established alongside gene transfer, because essential light sensing and regulatory systems were originally incorporated into plant cells by the photosynthetic endosymbiont. In this short article, we discuss the origins, early days and evolution of the plastid retrograde signal(s).

## INTRODUCTION

Eukaryotic cells contain various organelles, and continuous monitoring of and responding to their status are critical functions of the nucleus for managing cell integrity. Thus, eukaryotic cells have evolved intricate signaling mechanisms from the nucleus to organelles as well as from organelles to the nucleus (Woodson and Chory, 2008). Among such organelles, mitochondria and chloroplasts originated from symbioses with ancient bacteria, and show particular complexity in their structure and related signaling processes. Most notably, they contain their own genomes that were inherited from the ancestral bacteria, and concomitantly, bacterial-type gene expression and other cellular machineries are present in each organelle, independent of the nuclear system (Barbrook et al., 2010). These points discriminate these organelles from others, and this article mainly deals with chloroplasts.

Since the majority of chloroplast proteins are encoded by the nuclear genome and targeted into chloroplasts after translation in the cytoplasm (Bock and Timmis, 2008), communication in the nucleus-to-organelle direction is easily understandable (anterograde signaling). However, organelle-to-nucleus signaling, named retrograde signaling, has been the subject of controversy despite decades of research. In plant cells, changes in organelle condition such as oxidative damage or redox status have been known to modulate nuclear gene expression (Apel and Hirt, 2004; Pfannschmidt et al., 2009). Thus, genetic and other cell biological approaches have been used to clarify the relevant signaling pathway, and a number of genetic and metabolic components, as well as multiple signaling pathways, have been identified (Inaba et al., 2011). However, it should be noted that most of this research has been concentrated on the pathways modulating nuclear gene expression, and the non-transcriptional aspects of retrograde signaling have not been extensively examined thus far. We recently proposed that in a primitive red alga, a plastid-derived tetrapyrrole signal works to coordinate organelle DNA replication (ODR) and nuclear DNA replication (NDR), independent of transcriptional regulation in the nucleus (Kanesaki et al., 2009; Kobayashi et al., 2009, 2011). Here, we discuss early days of plastid retrograde signaling from the standpoint of its origin and evolution.

## COORDINATION OF DNA REPLICATION IN PLANT CELLS

In plant cells, three independent organelles – chloroplasts, mitochondria, and the nucleus – each contain their own genome. While only one nucleus is usually present in a cell, dozens of chloroplasts and mitochondria are found in most land plant cells. The proliferation cycle of plant cells is defined based on the S and M phases, which correspond to NDR and cell division, respectively, as in other eukaryotic cells. Similarly, chloroplasts and mitochondria proliferate by replication of their own genomes and subsequent division, and are inherited by daughter cells through division of the cytoplasm. However, abundantly present chloroplasts and mitochondria asynchronously proliferate throughout the cell cycle phases of higher plant cells (Birky, 2001), and not only division but also fusion is frequently observed for mitochondria (Logan, 2006, 2010). Thus, the interrelationship between the cell cycle and the organelle proliferation cycles remains unclear in plant cells.

Microscopic analyses of ODR and NDR during leaf and root development have shown that ODR is extensively activated during the early phase of the development of these organs, while NDR appears to occur after the ODR activation phase (Sakai et al., 2004). These observations indicate that ODR is not strictly co-regulated with NDR, but has some flexible links to NDR by unknown signaling mechanisms. In addition, a recent report strongly suggested links between the chloroplasts’ developmental status and NDR during leaf development; Andriankaja et al. (2012) reported that differentiation of the chloroplast photosynthetic machinery is important for cell cycle arrest and the onset of endoreduplication and cell expansion during leaf development in *Arabidopsis*. The authors suggested that some retrograde signal from chloroplasts might be deeply involved in the cell cycle phase transition.

Organelle DNA replication precedes NDR in plant cells; this tendency is most evident in unicellular red algae of Cyanidiales such as *Cyanidium caldarium* and *Cyanidioschyzon merolae*, which contain only one chloroplast and one mitochondrion per cell. In these cells, ODR in the chloroplast and mitochondria occurs after the onset of the cell cycle, and NDR always occurs after ODR. Particularly unique among the unicellular red alga, *Cyanidioschyzon merolae* performs binary fission, and the division of each organelle occurs only once in a cell cycle (Suzuki et al., 1994). In addition, complete genome sequences of the nucleus, chloroplast, and mitochondrion are now available (Ohta et al., 1998, 2003; Matsuzaki et al., 2004), and tools for molecular genetic analyses have been extensively developed in *Cyanidioschyzon merolae* (Ohnuma et al., 2008, 2009; Imamura et al., 2010). We thus analyzed the interconnection between ODR and NDR using this model cell system. As the results have already been published in two papers (Kobayashi et al., 2009, 2011), we only summarize the main points here:

Under dark conditions, the cell cycle of *Cyanidioschyzon merolae* is arrested at the G1 phase. Initiation of the S phase requires activation of a cyclin/cdk protein kinase complex, Cyclin1/CDKA, but Cyclin1 is continuously recognized by an F-box protein (Fbx3) of ubiquitin ligase in darkness, which results in ubiquitination and degradation of Cyclin1 by proteasome and prevention of NDR. Light illumination activates the cell cycle and prompts entry into the S phase, however, it is ODR not NDR that is primarily activated by the light. Activation of ODR somehow results in accumulation of Mg-protoporphyrin IX (Mg-ProtoIX) very likely in the cytosol, and subsequent interaction of Mg-ProtoIX with Fbx3 prevents the ubiquitination and degradation of Cyclin1, which results in Cyclin1 accumulation, Cyclin1/CDKA activation, and finally NDR activation. In this working model, Mg-ProtoIX is produced only in the chloroplast, and thus can be considered a *bona fide *retrograde plastid signal. In the case of nuclear gene expression control, Mg-ProtoIX has long been suggested as a plastid retrograde signal (Nott et al., 2006). However, recent reports in the field of plastid retrograde signaling have denied the specific role of Mg-ProtoIX in the nuclear transcriptional regulation of vascular plants (Mochizuki et al., 2008; Moulin et al., 2008). In any case, the involvement of the tetrapyrrole biosynthetic pathway itself is likely, and it is intriguing to consider why these compounds are critical to interorganellar communication.

**FIGURE 1:**
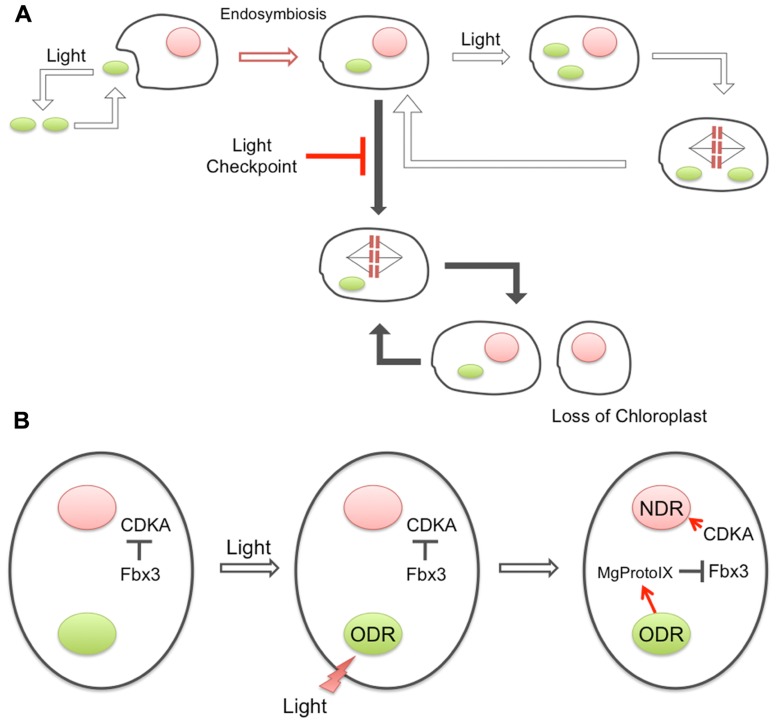
**Evolution of the coupling between ODR and NDR**. **(A)** Prior to endosymbiosis, the ancestral cyanobacteria were living phototrophically and dependent on light, while the host eukaryotic cells were proliferating independent of light conditions. After the engulfment, the primitive chloroplast would have required light for proliferation, while the host did not. Therefore, the uniquely acquired chloroplast may have been lost if the host cell could proliferate in the dark. A newly evolved checkpoint system, “light checkpoint” in the figure, prevented host proliferation in the dark to maintain the endosymbiotic association. **(B)** Architecture of the light checkpoint. Under dark conditions, an F-box component of E3-ubiquitin ligase, Fbx3, recognizes Cyclin1, resulting in ubiquitination and prompt degradation. Cyclin1 is the cyclin that forms a complex with CDKA and activates NDR, and thus, degradation of Cyclin1 prevents NDR in the dark. Light illumination somehow activates ODR and results in the accumulation of Mg-ProtoIX in the cytosol. Interaction of Mg-ProtoIX with Fbx3 prevents the ubiquitination of Cyclin1 and induces the activation of CDKA and NDR.

Prior to endosymbiosis, the cell cycle of the host eukaryote was probably non-phototrophic and likely driven by the usual Cyclin/CDK system that is unrelated to the external light conditions for photosynthesis. However, the cyanobacterial endosymbiont must have required light for proliferation. Thus, the endosymbiont of the early photosynthetic eukaryote was likely to be lost under dark conditions, unless some mechanism to couple the host–symbiont proliferation cycles evolved (**Figure 1**). The mechanism for the ODR–NDR coupling described above requires basically only one specific protein, Fbx3, to be established as an additional cytoplasmic component. Providing that the evolution of Fbx3 adequately explains the chloroplast-to-nucleus DNA replication coupling, this mechanism could have been established at a very early phase of chloroplast evolution because of its extreme simplicity. In addition, it should be noted that this mechanism does not require any gene transfer events from the chloroplast to the nucleus. Since the well-known plastid retrograde signals are related to the transcriptional regulation of nuclear genes encoding chloroplast-targeted proteins, gene transfer events are prerequisite for their existence. The establishment of endosymbiosis would have been prior to such gene transfer events, and our recent findings may have supported the stabilization of the transition states. A similar coupling mechanism has also been suggested to be present in vascular plant cells (Kobayashi et al., 2009), again indicating its common and early origin during plant evolution.

## LIGHT-RESPONSIVE TRANSCRIPTIONAL REGULATION IN THE NUCLEUS

The significance of the coordination of nuclear and chloroplast DNA replication, which could have been established during very early stages of chloroplast evolution after the endosymbiotic event, is described above. In addition to DNA replication, along with gene transfer from the chloroplast to the nuclear genome, light-dependent expression of genetic information should also be coordinated between the nucleus and chloroplasts to enable suitable stoichiometry of gene products that are encoded in separate genomes (Goldschmidt-Clermont, 1998; Woodson and Chory, 2008). In vascular plants, it has been speculated that such coordination could be mediated by bi-directional (anterograde and retrograde) signaling pathways (Pogson et al., 2008; Barajas-López et al., 2012), and additionally, various types of photoreceptors as well as light signaling modules are well-known to regulate the light-dependent expression of a set of nuclear genes (Jiao et al., 2007). However, it is very difficult to imagine how and when this coordination and/or light regulation of nuclear gene expression could have been established in the course of chloroplast evolution after the endosymbiosis with an ancient cyanobacterial cell.

**FIGURE 2:**
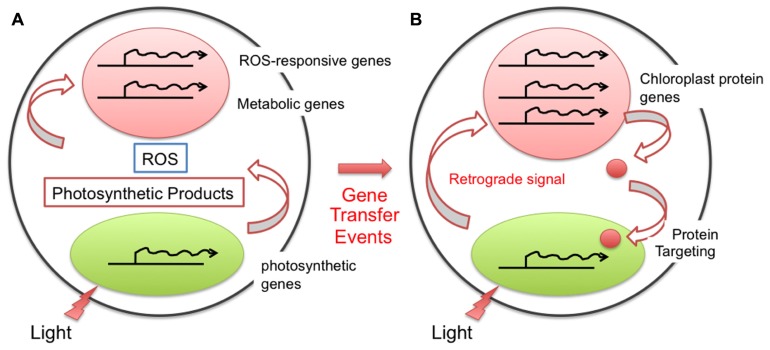
**Evolution of the chloroplast retrograde signal for nuclear transcriptional regulation**. After endosymbiosis, the chloroplast performed photosynthesis, generating photosynthetic metabolites as well as reactive oxygen species (ROS). The ability to sense and respond to these compounds in the cytoplasm should have been present even prior to the gene transfer event, and thus the nucleus was likely able to cope with them without any specific evolution **(A)**. After the gene transfer event from the chloroplast to the nucleus, the location of the gene and its function differentiated. To modulate the function and amount of the gene product properly, the cell evolved a regulatory loop through a specific retrograde signaling pathway **(B)**.

Before endosymbiosis, an ancient eukaryotic cell with a nucleus and mitochondria should have some, but not so highly complicated light-responsive regulation of gene expression because it was unnecessary to respond to the light environment as a non-photosynthetic organism. Conversely, cyanobacteria, which require oxygenic photosynthesis for their survival, should have a set of photosensory and photoregulatory mechanisms. Just after endosymbiosis, during the early stages of chloroplast evolution, light-responsive gene expression derived from cyanobacteria must have been completed inside the chloroplast (symbiont), and the regulation of nuclear gene expression should have been irrespective of the light conditions. However, mutual coordination of various cellular parameters, including metabolic fluxes, must have become crucial between the chloroplast and the host cell (**Figure 2**). In addition, gene transfer from the chloroplast to the nuclear genome has gradually occurred. Therefore, in a later phase of chloroplast evolution, light signals should have become transmitted into the nucleus to establish light-responsive coordinated gene expression as a photosynthetic eukaryote. During the subsequent evolution, plant-type photoreceptors and light signaling pathways were likely established.

Recently, we hypothesized a primitive mechanism for light response, as well as light regulation of nuclear gene expression, based on the analysis of a primitive red alga, *Cyanidioschyzon merolae*. Light-dependent transcriptional induction in the nucleus has been shown to occur in *Cyanidioschyzon merolae* (Fujiwara et al., 2009). However, no typical plant-specific photoreceptors (phytochromes and phototropins) were encoded in the *Cyanidioschyzon merolae* genome (Matsuzaki et al., 2004), although a group of cryptochrome-like genes are found (Asimgil and Kavakli, 2012). These lines of evidence suggest that a part of the photosensing in *Cyanidioschyzon merolae* occurs inside the chloroplast and thus light signal(s) could be transmitted into the nucleus by some retrograde mechanisms. As a candidate mediator for chloroplast-to-nucleus signaling in vascular plant cells, Mg-ProtoIX (which has already been mentioned in the previous section as a possible regulator for coordination of DNA replication between nuclear and organelle genomes) was examined to determine whether it could also regulate nuclear gene expression in *Cyanidioschyzon merolae*. As is consistent with recent negative reports for this signaling molecule (Mochizuki et al., 2008; Moulin et al., 2008), we could not obtain any experimental evidence supporting this hypothesis (Kanesaki et al., 2009). These observations also suggest the presence of another, presumably more primitive but fundamental, signaling pathway in *Cyanidioschyzon merolae*, which can transduce light signals into the nucleus (Kanesaki et al., 2012).

In bacteria, fungi, and plants, it is well-known that two-component regulatory systems are involved in the sensing of, and regulatory responses to, various environmental stimuli. This system is normally composed of sensory histidine kinases (HKs) that perceive environmental signals and response regulators required for output regulation such as transcriptional regulation (Jung et al., 2012). There are genes for one histidine kinase (CmHIK) and two response regulators (Ycf27 and Ycf29) in the *Cyanidioschyzon merolae* nuclear and chloroplast genomes, respectively (Ohta et al., 2003; Matsuzaki et al., 2004). They are possibly involved in light-responsive transcriptional regulation in chloroplasts because their orthologous factors in cyanobacteria are considered to be required for light responses (Ashby and Mullineaux, 1999; Ashby et al., 2002). Since these factors and systems are not conserved in the chloroplasts of vascular plants, it is natural to consider that *Cyanidioschyzon merolae* has a more chloroplast-autonomous signaling mechanism, especially in response to light. This could represent a primitive style of light response during the course of chloroplast evolution. As mentioned above, the transcription of most nuclear genes is also regulated in a light-dependent manner in *Cyanidioschyzon merolae*. Thus, in the early days of chloroplast evolution, we hypothesize that a chloroplast photosensory mechanism(s), such as the two-component system inherited from cyanobacteria, might also have been responsible for light-dependent nuclear transcription via an unidentified chloroplast-to-nucleus retrograde signaling pathway(s). The establishment of a complex coordination mechanism for nuclear and organelle gene expression in response to light environments likely required a long time period. However, light-dependent nuclear transcription itself might have evolved alongside gene transfer to the nucleus. This retrograde signal(s) could be partly derived from chloroplast photosensory components including the two-component system, as well as an event of photosynthetic electron transfer. In any case, during early phases of chloroplast evolution, we expect that the development of fundamental crosstalk between the nucleus (host) and the chloroplast (symbiont) was essential to effectively respond to light environments, and to coordinate DNA replication and transcription of both genomes.

## Conflict of Interest Statement

The authors declare that the research was conducted in the absence of any commercial or financial relationships that could be construed as a potential conflict of interest.
